# Household costs associated with seeking malaria treatment during pregnancy: evidence from Burkina Faso and The Gambia

**DOI:** 10.1186/s12962-022-00376-x

**Published:** 2022-08-20

**Authors:** Laetitia Duval, Elisa Sicuri, Susana Scott, Maminata Traoré, Bunja Daabo, Halidou Tinto, Koen Peeters Grietens, Umberto d’Alessando, Henk Schallig, Petra Mens, Lesong Conteh

**Affiliations:** 1 Centre d’Economie de La Sorbonne, University Paris 1 Panthéon-Sorbonne, Paris, France; 2 Department of Health Policy, London School of Economics and Political Science, London, UK; 3 ISGlobal, Barcelona Centre for International Health Research, Hospital Clinic, Universitat de Barcelona, Barcelona, Spain; 4 Infectious Disease Epidemiology Department, London School of Hygiene & Tropical Medicine, London, UK; 5 Medical Research Council Unit The Gambia at London, School of Tropical Medicine and Hygiene, Fajara, The Gambia; 6 Institut de Recherche, en Sciences de La Santé - Clinical Research Unit of Nanoro, Nanoro, Burkina Faso; 7 Medical Anthropology Unit, Department of Public Health, Institute of Tropical Medicine, Antwerp, Belgium; 8 Department of Medical Microbiology, Parasitology Unit, Academic Medical Centre, Amsterdam, The Netherlands

**Keywords:** Malaria, Pregnancy, Cost, Remittances, Sub-Saharan Africa, Burkina Faso, Gambia

## Abstract

**Background:**

Malaria in pregnancy remains a major health threat in sub-Saharan Africa to both expectant mothers and their unborn children. To date, there have been very few studies focused on the out of pocket costs associated with seeking treatment for malaria during pregnancy.

**Methods:**

A cross-sectional survey was undertaken in Burkina Faso and The Gambia to estimate the direct and indirect costs associated with outpatient consultations (OP) and inpatient admissions (IP). Direct costs were broken down into medical (admission fees, drug charges, and laboratory fees), and non-medical (transportation and food). Indirect costs reflected time lost due to illness. In total, 220 pregnant women in Burkina Faso and 263 in The Gambia were interviewed about their treatment seeking decisions, expenditure, time use and financial support associated with each malaria episode.

**Results:**

In Burkina Faso 6.7% sought treatment elsewhere before their OP visits, and 27.1% before their IP visits. This compares to 1.3% for OP and 25.92% for IP in The Gambia. Once at the facility, the average direct costs (out of pocket) were 3.91US$ for an OP visit and 15.38US$ of an IP visit in Burkina Faso, and 0.80US$ for an OP visit and 9.19US$ for an IP visit in The Gambia. Inpatient direct costs were driven by drug costs (9.27US$) and transportation costs (2.72US$) in Burkina Faso and drug costs (3.44 US$) and food costs (3.44 US$) in The Gambia. Indirect costs of IP visits, valued as the opportunity cost of time lost due to the illness, were estimated at 11.85US$ in Burkina Faso and 4.07US$ in The Gambia. The difference across the two countries was mainly due to the longer time of hospitalization in Burkina Faso compared to The Gambia. In The Gambia, the vast majority of pregnant women reported receiving financial support from family members living abroad, most commonly siblings (65%).

**Conclusions:**

High malaria treatment costs are incurred by pregnant women in Burkina Faso and The Gambia. Beyond the medical costs of fees and drugs, costs in terms of transport, food and time are significant drivers. The role of remittances, particularly their effect on accessing health care, needs further investigation.

## Background

Malaria in pregnancy (MiP) is a major public health problem in sub-Saharan Africa (SSA) [1]. It also places an economic toll on individuals and nation states [2]. Pregnant women are at higher risk of malaria than other adults, leading to potentially adverse outcomes for their foetus, newborn child and themselves [3–6] which can also have long term economic consequences. In 2018, prevalence of malaria infection in pregnancy was highest in the West African subregion and Central Africa (each with 35%) [1]. The World Health Organization (WHO) advocates prompt and effective management of clinical cases, long-lasting insecticidal nets and intermittent preventive treatment during pregnancy (IPTp). In all areas with moderate to high malaria transmission in Africa, a full therapeutic course of the antimalarial sulfadoxine-pyrimethamine (IPTp-SP) should be systematically given to pregnant women at each routine antenatal visit as far as these are at least one month apart and regardless of infection status [7, 8]. On average, only 31% of pregnant women across thirty-six African countries received the recommended three doses of IPTp in 2018. Burkina Faso reached IPTp coverage of more than 50% and in The Gambia the estimated coverage was 30% [1].

The success of IPTp delivery depends partly on access to, and use of, antenatal care (ANC) by pregnant women. In 2016 about 26% of women did not attend ANC facilities during their pregnancy [9]. It is essential, therefore, to understand barriers to accessing facility-based care if both IPTp coverage and rates of pregnant women receiving prompt and effective treatment are to increase.

A range of geographic, economic, social and cultural factors contribute to the low use of antenatal clinics. These factors include, among others, distance and poor road infrastructure, low level of education, poverty, limited access to information or traditional values [10, 11]. At the health facility level, confusion about timing of IPTp and difficult assessment of the gestational age have also been identified as obstacles [12, 13]. Costs, both direct and indirect, have been shown to be an important barrier to pregnant women’s use of health facilities, both for routine antenatal visits and to seek treatment more generally.

Costing studies associated with MiP are few in number compared to other malaria interventions [6, 14] and are largely in the form of economic evaluations reporting either the actual or projected costs and cost-effectiveness of preventive interventions such as IPTp and nets [15–19]. Few evaluate costs from the provider [15, 18, 20, 21] and/or user perspectives [2, 18, 21–24] with only one other African study calculating treatment costs for malaria while pregnant [2]. The aim of this study was to estimate the costs of malaria episodes incurred by pregnant women in Burkina Faso and The Gambia using primary, individual-level data collected after an outpatient consultation or at discharge after hospitalization. Beyond the costs, the study also provides an opportunity to explore treatment seeking behaviour in terms of identifying the range of places care was sought for an individual malaria episode. While we acknowledge that the costs of preventing and treating MiP are largely borne by the health care providers of these countries, pregnant women are likely to incur costs that can prohibit accessing prompt and effective health care. We explore issues of access, in part, by exploring how women in both study settings reach their health facilities, both in terms of the direct costs of doing so (transport payments) and the indirect costs (using time as a proxy). To our knowledge, these are the first published adult treatment seeking cost estimates for malaria in either Burkina Faso or The Gambia.

While our focus is on the costs of seeking treatment for malaria while pregnant, we also asked some explorative questions about financial support the respondents may have received. We were particularly interested in the frequency and sources of remittances. Evidence shows that remittances improve various health outcomes [25–28]. The extent remittances feature in these settings and amongst pregnant women, has not been explored. While our study was not designed to infer any relationships between financial support and health outcomes, it provided the first insights into the extent and type of financial support flowing into the households of pregnant women in both settings.

## Methods

### Study settings and populations

The costing study was ancillary to the cluster randomized trial called “Community-based scheduled screening and treatment of malaria in pregnancy for improved maternal and infant health: a cluster-randomized trial in The Gambia, Burkina Faso and Benin” (commonly referred to as COSMIC study). The study aimed to determine whether adding community-based scheduled screening and treatment of malaria (CSST) by community health workers (CHWs) to standard IPTp-SP would further reduce placental malaria compared to IPTp-SP alone. [29] In practical terms CSST differed from routine care in two ways: (1) CHWs in the intervention arm were trained in malaria case management and malaria in pregnancy and asked to encourage pregnant women in their catchment areas to attend antenatal care as early as possible; (2) After their initial antenatal care visit, each pregnant woman in the intervention arm received monthly home visits from a CHWs which included giving an RDT and collecting a blood slide, regardless of malaria symptoms, up to the last week of gestation. All malaria positive women received the antimalarial artemether-lumefantrine. Severely ill women were referred to the health centre for further care [29].

Between November 2013 and November 2015, a total of 4731 pregnant women across the three countries took part in the 2-arm cluster-randomized, controlled trials to assess the clinical outcomes. The cross-sectional costing study presented here was conducted between August 2014 and December 2015 and designed to assess the direct and indirect costs associated with a single malaria episode from the pregnant women’s perspective in all three countries. However, data from Benin was excluded because the clinical trial became subject to rumours and accusations of placenta being sold for mystical and financial gain by staff despite community sensitization meetings and standardized informed consent procedures. After discussions with the data and safety monitoring board and the local ethics committee, it was decided to stop the data collection in this study site [30].

In The Gambia, the study was conducted in the eastern part of the country on the southern bank of the Upper River Region (about 170,000 inhabitants), around the Basse Health Centre and satellite health facilities. Although malaria in The Gambia has declined significantly over the last 10–15 years, there is still moderate and highly seasonal (July–December) transmission in its eastern region. Despite reductions in malaria, about 15% of pregnant women are still shown to have placental malaria at delivery [4]. In The Gambia, pregnant women with suspected malaria or other health concerns are not expected to pay a fee for hospitalization or outpatient visits. In Burkina Faso, the study was conducted in the Centre-West of the country, in the Nanoro Health District, catchment area (about 145,000 inhabitants), where malaria transmission is high and extremely seasonal (June-December). Studies in Burkina Faso have estimated prevalence of malaria infection amongst pregnant women at between 15.7% and 18.1% [31, 32]. In Burkina Faso, at the time of the trial, pregnant women were expected to pay for malaria treatment.

### Data collection

Prior to starting the COSMIC study, community sensitization was carried out at each study site. Following cultural norms in both settings, consent was sought at the community level through discussions with village elders. Where appropriate further community meetings were then held to explain the study objectives to all community members. Experienced data collectors explained in the local language the written information and consent forms to potential participants. This conversation outlined study goals, the topic and type of questions respondents could expect and highlighted their right to decline participation, to interrupt or withdraw from the questionnaire at any time without giving a reason. If an individual agreed to answer the costing questionnaire, they signed the informed consent (by thumb print and the signature of an independent witness in case of illiteracy). The economic protocol followed the same inclusion and exclusion criteria as that used in the clinical trial. [33] Pregnant women resident in the study area were invited to participate in the costing study before leaving a health facility. Women with a history of sensitivity to sulphonamides and vulnerable persons (for example, the mentally impaired) were excluded. In The Gambia, pregnant adolescents younger than 16 years were not enrolled unless consent was given by a responsible adult. In Burkina Faso, a pregnant woman was considered an adult if married (regardless of age).

For those who consented, structured questionnaires were administered. The questionnaires comprised of five sections: 1. socio-demographic characteristics of the study participants such as age, level of education and receipt of money from anyone living abroad; 2. transportation costs to the health facility; 3. treatment seeking behavior and previous treatments for symptoms associated with the same malaria episode; 4. direct costs of treatment which was confirmed with data abstracted from the prescription orders; and 5. time lost because of the illness. See Annex 1–4 for English and French versions of the questionnaire.

Costs were broken down into both direct and indirect costs. Direct costs (out-of-pocket expenses) were further broken down into medical (such as admission fees, drug charges, and laboratory fees), and non-medical (such as transportation and food). Indirect costs reflected time lost because of the illness. Indirect costs were collected for inpatient visits only. They were calculated by multiplying reported time lost by the nominal value of the median monthly permanent income per capita of households in Burkina Faso and The Gambia, represented by country specific estimates by the International Labour Organisation [22, 24, 34].

### Data management and analysis

All data were collected through standardized questionnaires, double entered into a specially designed database and verified. Analysis was conducted using Microsoft Excel and Stata software (version 14, College Station, Texas, USA). All questionnaires were labelled with the pregnant woman’s unique identity number and date of collection, thus ensuring anonymity. Costs were collected in local currencies.

Missing values were rare, never beyond the 3% of a variable, and reflected information that participants did not want to disclose or could not remember. Our values can, therefore, be interpreted as a lower bound of the estimated categories.

Costs were then converted to US dollars (US$) using an average exchange rate for 2016 [35]. In most instances, using this currency conversation approach, we present findings in their 2016 monetary value in current absolute terms. In Table 2, we also present costs in international dollars (INT$) having accounted for World Bank purchasing power parity conversion rates [33]. This approach allows for further cross country comparison as it takes into account the standards of living between countries based on a ‘basket of goods’.

## Results

Three hundred fifty-five outpatients (150 in Burkina Faso and 155 in The Gambia) and 178 inpatients (70 in Burkina Faso and 108 in The Gambia) were interviewed (Table 1).

**Table 1:** Descriptive characteristics of the respondents

Country	Burkin Faso		Gambia	
	Out patient	In patient	Out patient	In patient
Total number of observations^a^	150	70	155	108
Mean Age in years (SD)	24 (5.80)	24 (5.55)	27 (5.80)	27 (5.12)
Mean Number of children (SD)	1.43 (1.72)	1.36 (1.68)	1.75 (1.69)	1.75 (1.77)
	Percentage Breakdowns of Characteristics^b^			
**Diagnosis**				
Clinical malaria	100	71	65	56
Severe anemia	0	16	35	39
Malaria & severe anemia	0	13	0	5
**Education**				
None	77	73	65	70
Primary	12	11	21	18
Secondary	11	14	14	12
**Primary Religion**				
Catholic	53	53	2	4
Islam	33	36	97	96
Traditional African	14	11	1	0
None	1	0	0	0
**Ethnicity**				
Mossi	92	79	–	–
Gourounsi	5	9	–	–
Peuhl	3	11	–	–
Mandinka	–	–	30	41
Fula	–	–	37	27
Serahuleh	–	–	30	28
**Marital status**				
Married	86	73	92	93
In relationship	12	24	1	2
Single	1	3	6	6
Separated	1	0	1	0
**Activity**				
Housewife	29	41	15	11
Farmer	55	36	54	65
Market trader	6	9	6	4
No occupation	6	11	9	6
Others	4	3	15	14
**Partner’s activity**				
Farmer	68	57	67	81
Market trader	9	6	12	6
Civil servant	3	3	13	8
No occupation	0	1	1	0
Others	21	33	7	5

*SD* Standard deviation

^a^Total number of observations refers to the total sample size for this study. For certain variables we had missing values, although never beyond the 3%.

^b^Rounded to the nearest percentage point

### Outpatient characteristics

Pregnant women were younger and had on average less children in Burkina Faso than in The Gambia. In addition, there was a higher proportion of women with no formal education in Burkina Faso (76.6%) than in The Gambia (64.5%), with only 10.6% of women having completed secondary education in Burkina Faso and 13.5% in The Gambia. In both countries, the main activity was farming, for the women (54.6% in Burkina Faso and 54.1% in The Gambia) and their partners (68.0% in Burkina Faso and 66.9% in The Gambia). About half (52.7%) of the women in Burkina Faso were Catholics while most (97.4%) Gambian women were Muslims.

### Inpatient characteristics

Inpatient characteristics were similar to outpatient ones. Women in The Gambia were older (27.02 years) and had a higher number of children (1.75) than those in Burkina Faso (23.87 years and 1.47). Similarly, most women were farmers and had no formal education.

### Total costs

#### Outpatient visit costs

Table 2 reports the outpatient visit costs broken down by all cost centres. Medical costs including lab and other fees and drug costs were significantly lower in The Gambia (0.80US$) than in Burkina Faso (3.57US$). The outpatients in both countries did not report cost for food. For outpatients, direct costs were statistically different between the two countries (Pvalue = 0.00). Indirect costs were not collected for outpatient visits.

**Table 2:** Unit costs of inpatient and outpatient visits (US$ &Int$, 2016)

Country	Burkina Faso		The Gambia		
	Out patient	In patient*	Out patient		In patient
Number of observations ‘seeking previous treatment’ compared to total sample surveyed	10/150	19/70	2/155		28/108
**Previous treatment seeking cost**					
Total median direct cost of previous treatment (US$)^b^	2.04 (1.02; 2.72)^a^	0.34 (0; 4.59)	0.86 (0.57;1.14)		1.72 (1.14;3.44)
Total median direct cost of previous treatment (Int$)^c^	6.01 (3.00;8.01)	1.00 (0;13.52)	2.76 (1.83;3.65)		5.51 (3.65;11.03)
**Direct costs**					
Median cost lab and other test fees	0.17 (0; 0.34)	1.53 (0; 5.27)	0.80 (0.57;3.44)		0.57 (0.57; 1.14)
Median cost drugs	3.4 (2.55; 4.08)	9.27 (7.14; 13.00)	0 (0;1.83)		3.44 (2.75;4.02)
Median cost transportation	0 (0;1.7)	2.72 (0; 5.44)	0 (0;2.29)		2.06 (0; 2.75)
Median cost food	NA	2.04 (1.36; 3.4)	NA		3.44 (2.75; 3.44)
Total median direct costs	3.91 (2.93; 5.61)	15.38 (10.54;20.31)	0.80 (0.57;3.90)		9.19 (7.12;11.03)
Total median direct costs (Int$)	11.51 (8.63;16.52)	45.29 (31.04;59.81)	2.62 (1.83;12.50)		29.46 (22.82;35.36)
**Indirect costs**					
Median value of time lost because of the illness (US$)	NA	11.85 (7.11; 16.59)	NA		4.07 (4.07; 5.43)
Median value of time lost because of the illness (Int$)	NA	34.90 (20.94;48.86)	NA		13.05 (13.05;17.41)

*NA* Not asked

^a^Interquartile Range in parenthesis

^b^US$ conversion Rates were based on 2016 annual exchange rates Burkina Faso was 0.0017 and The Gambia was 0.02299

^c^Int$ 2016 were based on PPP conversion factors private consumption (Local Currency Unit per international dollar) from the World Bank. Burkina Faso was 199.74 and The Gambia was 13.57

#### Inpatient visit costs

Table 2 reports also the inpatient visit costs. Medical costs for inpatients were higher in Burkina Faso (10.80US$) than in The Gambia (4.01US$). Although, the cost of food was higher in The Gambia (3.44US$) than in Burkina Faso (2.04US$), direct costs were significantly higher in Burkina Faso (15.38 US$) than in The Gambia (9.19US$). Indirect costs, including the value of time lost because of the illness, were significantly higher (11.85US$) in Burkina Faso than in The Gambia (4.07US$), a difference explained by the longer average hospitalization in Burkina Faso (3.55 days) than in The Gambia (1.21 days).

### Treatment seeking behavior and associated costs prior to facility visit

Table 3 reports the sample of outpatients and inpatients who sought care elsewhere before consenting to answer our questionnaire. Seeking treatment for the current malaria episode before the OP visit was uncommon: 6.7% in Burkina Faso and 1.3% in The Gambia. Indeed, in The Gambia only two women (1 from home and 1 from a pharmacy) sought treatment before their OP visit while, in Burkina Faso, 3 women had treatment from home, 2 from pharmacies, and 5 from a hospital). The mean cost of this first treatment was 1.51US$ in Burkina Faso and 0.86US$ in The Gambia. Only outpatients in Burkina Faso (N = 3) reported having sought treatment from a second place, at a mean cost of 4.87US$.

**Table 3:** Frequency and costs of previous treatment(s) sought

Country	Burkina Faso					Gambia			
	Out patient			In patient^a^		Out patient		In patient	
	%		N	%	N	%	N	%	N
Any treatment before	6.67		10/150^b^	27.14	19/70	1.30	2/155	25.92	28/108
***Places previous treatment sought before survey***									
**First place treatment sought**									
Home	30.00		3	43.47	10	50.00	1	32.14	9
Traditional healer	0.00		0	4.34	1	0.00	0	10.71	3
Market	0.00		0	0.00	0	0.00	0	0.00	0
Pharmacy	20.00		2	17.39	4	50.00	1	35.71	10
Hospital	50.00		5	17.39	4	0.00	0	21.42	6
**Second place treatment sought**									
Home	50.00		2	25.00	1	0.00	0	0.00	0
Traditional healer	25.00		1	0.00	0	0.00	0	0.00	0
Market	0.00		0	0.00	0	0.00	0	0.00	0
Pharmacy	0.00	0		0.00	0	0.00	0	100.0	1
Hospital	25.00	1		75.00	2	0.00	0	0.00	0
***Costs of previous treatment before survey (US$)***									
Mean cost of first treatment	1.51			2.21		0.86		2.74	
Mean cost of second treatment	4.87			20.14		0.00		0.00	
Mean total cost previous treatment	2.72			4.27		0.86		2.74	

^a^Note that ‘previous treatment seeking costs’ for inpatient costs includes any pre-admission outpatient costs incurred during the malaria episode.

^b^The sample size for ‘any treatment before’ represents the total number of respondents who had sought care from *at least one* sources before the time of the interview. Therefore 10/150 represents the 10 women out of all those interviewed as outpatients in Burkina Faso (n=150), who had been to at least one other provider prior to answering our questionnaire. Of those 10 respondents, 4 had also sought treatment at a second place

Before their IP visit, 27.1% and 25.9% of women sought treatment in Burkina Faso and The Gambia, respectively. Inpatients reported being ill on average 3.21 (range 1–20) days in Burkina Faso and 1.83 (range 1–4) days in The Gambia before being hospitalized. The mean cost of the first treatment for IP was higher in The Gambia (2.74US$) than in Burkina Faso (2.21US$).

The mean total cost for an OP consultation, including any previous treatment, was 2.72US$ in Burkina Faso and 0.86US$ in The Gambia. For hospitalization, the cost was 4.27US$ in Burkina Faso and 2.74US$ in The Gambia for an admission. For inpatients, costs for first treatment sought and total were statistically different between the two countries (Pvalue = 0.0023 and Pvalue = 0.0241, respectively). For outpatients, costs for first, second treatment sought and total were not statistically different between the two countries (Pvalue = 1.000, Pvalue = 0.6547 and Pvalue = 0.5163, respectively).

### Transportation to the health facility

Table 4 reports transportation costs. They are considered as direct non-medical costs. In Burkina Faso, most outpatients travelled by bicycle (52.6%) and motorbike (26.0%) while in The Gambia about half (51.61%) travelled by foot. The use of an ambulance for inpatients was slightly higher in Burkina Faso (5.71%) than in The Gambia (3.85%). The mean time taken to reach the health facility was similar in the two countries, around 24 min (range 2–90 min in Burkina Faso; range 1–60 min in The Gambia). Average reported travel time was similar across all modes of transport, between 25 and 35 min, with a longer average time (50 min) reported when visiting Burkina Faso outpatients on a bicycle.

**Table 4:** Transportation to the health facility

Country	Burkina Faso				Gambia			
	Out patient		In patient		Out patient		In patient	
	%	N	%	N	%	N	%	N^a^
**Transport type**								
On foot	21	32	9	6	52	48	8	2
Bicycle	53	79	17	12	0	0	0	0
Motorbike	26	39	67	47	14	13	4	1
Taxi-moto	0	0	0	0	14	13	35	9
Car	0	0	0	0	14	13	42	11
Donkey	0	0	0	0	6	6	8	2
Ambulance	0	0	6	4	0	0	4	1

	Time	N	Time	N	Time	N	Time	N
Time taken to reach the health facility (minutes)	24.70	150	34.77	70	24.15	155	36.33	108

^a^Rounded to the nearest percentage point

### Financial support

Table 5 reports additional information on financial support. In terms of health insurance, no pregnant woman in Burkina Faso had a health insurance while there were a few women with health insurance in The Gambia. Almost all women in The Gambia reported household savings while in Burkina Faso the proportion was extremely low. In The Gambia, a large part of household savings consisted of remittances sent by family members living abroad, with most of them made by siblings to the household (around 65%). Remittances were also made by other members of the family (27%). About half of the remittances sent to The Gambia came from Italy, followed by France (12.5%), Germany (9.6%), United States (6.7%) and United Kingdom (0.9%).

**Table 5:** Financial support

Country	Burkina Faso		Gambia	
	Out patient	In patient	Out patient	In patient
Number of observations	150	70	155	108
	Percentages^a^			
Health insurance	0	0	1	4
Savings in the household	1	3	90	96
Receiving government support	0	0	0	0
Money from abroad	5	1	88	96
Remitter				
Husband	3	1	6	4
Brothers/sisters	0	0	63	68
Children	0	0	1	0
Other family members	1	0	29	28
Friends	0	0	1	0
Country where remitter based				
Italy	0	0	38	58
Germany	0	0	12	10
France	0	0	19	13
United States	0	0	4	7
United Kingdom	0	0	9	1
Ivory Coast	5	1	0	0

^a^Rounded to the nearest percentage point

## Discussion

This study represented a rare opportunity to cost malaria treatment in two different settings, in Burkina Faso and The Gambia, from the perspective of pregnant women. We present the unit costs and cost breakdowns of both inpatient and outpatient, data that has not been available to date. We are able to show in Gambia treatment costs are lower, largely due to lower drug costs. Our data suggests that approximately 27% of pregnant women in both settings had sought care elsewhere before their inpatient visit, notably from home or the pharmacy. The vast majority of women in The Gambia reported financial support directed at their household, our study fell short of confirming if these remittances were used by the pregnant women towards health care.

The direct cost of inpatient treatment for malaria in Burkina Faso was US$15.38, the equivalent of 55% of the monthly female agricultural wage (estimated at US$28.31 per month [34]). In The Gambia it equated to 19% (using a monthly International Labour Organisation estimated female agricultural wage of US$49.35, compared to US$9.19 direct costs of inpatient care). We can therefore say with confidence that malaria treatment poses a significant economic burden on pregnant women in our study, particularly in Burkina Faso.

In 2016, a few months after the end of our data collection, the government of Burkina Faso established a free health care policy for women. The benefit package now covers a wide range of services including antenatal care, the prevention of anaemia and malaria. Consultation fees, prescriptions fees, laboratory tests, hospitalization expenses and the expenses of ambulance transportation between health facilities are also covered. A recent study showed that while the policy has provided effective financial protection, a significant proportion of women continue to pay for services and consumables that should be free of charge [36]. Our study reinforces the fact that even if there is no fee attached to health facility visits, there are still costs incurred around transport and food. This echoes a study published on the costs associated with malaria treatment among pregnant women in Colombia where transportation was a sizable part of outpatients’ and inpatients’ direct costs [21].

In Nigeria, the average cost of treating an episode of malaria during pregnancy was reported as US$11.86 (direct medical cost) and US$18.97 (direct nonmedical cost) [37]. While the unit costs are not directly comparable to ours due to methodological differences, the Nigerian study estimated annual total cost for malaria treatment during pregnancy of US$78.6 million (0.016% of the Nigerian Gross Domestic Product). The study emphasized that treatment costs in Nigeria are largely funded by out-of-pocket payments, further strengthening the argument to increase coverage of malaria prevention to help protect pregnant women against the financial strain of seeking treatment.

This is the first study to explore the frequency of financial support, more specifically remittances to households with pregnant women in both settings. While there appears little or no health insurance, in The Gambia most women received remittance from family members living abroad, mainly from a few European countries (Italy, France, Germany) and the United States. Estimates of the global Gambian diaspora range between 118,485 to 200,000, one of the highest rates of emigration in Africa, at approximately 9.2% of the population [38]. Our findings support 2019 World Bank estimates which state The Gambia, at 15.5%, was the second largest recipient of remittances as a proportion of its GDP in sub Saharan Africa [39]. In Burkina Faso, emigrants make up between 8 and 10% of the country’s population (with about 90% of these living in Côte d’Ivoire). Remittances as percentage of GDP for Burkina Faso in 2017 was much lower, at 3.39% [39, 40]. As stated previously, remittances are shown to improve health outcomes, however further work is needed, to determine if remittances are specifically associated with improved health outcomes for pregnant women and their newborns.

There are limitations to this study. The costs data were obtained from a cross-sectional survey. Ideally, a longitudinal data would allow us to estimate the economic burden of malaria over the course of the entire malaria season and/or individual pregnancies to better reflect the impact of multiple episodes. We were only able to capture the costs of malaria episodes from women who visited the health facilities included in the study. There is always the risk of the Hawthorne effect whereby the pregnant women might have modified aspects of their behaviour in response to their awareness of being part of a trial, but this is unlikely, given the trial offered no additional benefits in terms of access to care or finances in either trial arm. Finally, the costs in this study are associated with the immediate malaria infections in pregnant women and do not consider the long-term costs of treating the consequences of maternal infection on the infant. For instance, the consequences of low birth weight have been studied previously [41–43].

A recent review of the status of MiP highlights three main economic issues than need further attention. First, MiP accounts for a notable proportion of the total health-care budget; second, direct and indirect costs incurred by pregnant women for malaria prevention and treatment are high and; third, strategies to decrease costs incurred (e.g., vouchers, social marketing, and delivery through community approaches) are effective, but they need to be scaled up [6]. In this study, we address the second issue, by adding to the limited available information on treatment costs associated with MiP from the pregnant women’s perspective.

The key message to policy makers emanating from this study is the same in both countries; reaching pregnant women in rural areas and encouraging them to engage with ANC services early during their pregnancy is essential for malaria prevention and control as direct and indirect treatment costs associated with malaria episodes amongst this vulnerable group are high even if many services are officially provided for ‘free’ to pregnant women.

## Conclusions

Our results revealed the high costs incurred by pregnant women in Burkina Faso and in The Gambia. Even when accounting for the removal of user fees, the indirect costs of transport and food can be significant. The costs incurred by pregnant women may have a large impact on the budgets of households, and consequences on the allocation of their limited resources. Our findings also suggest that the role of remittances, particularly in The Gambia, needs further investigation with a focus on the effect they may have on accessing health care and improving health outcomes.

## Data Availability

The datasets used and/or analysed during the current study are available from the corresponding author on reasonable request.

## Abbreviations

ANC: Antenatal care
CHW: Community health worker
COSMIC: Community based scheduled screening and treatment of malaria in pregnancy for improved maternal and infant health
CSST: Community-scheduled screening and treatment
IP: In-patient
IPTp: Intermittent preventive treatment during pregnancy
IPTp-SP: Intermittent preventive treatment during pregnancy with sulfadoxine-pyrimethamine
OP: Out-patient
RDT: Rapid diagnostic test
SSA: Sub-Saharan Africa
WHO: World Health Organization
